# Influence of cutting velocity on surface roughness during the ultra-precision cutting of titanium alloys based on a comparison between simulation and experiment

**DOI:** 10.1371/journal.pone.0288502

**Published:** 2023-07-21

**Authors:** Yonggou Lou, Lei Chen, Hongbing Wu, Sandy To

**Affiliations:** 1 School of Haitian, Ningbo Polytechnic, Ningbo, China; 2 Ningbo JoysonQuin Automotive Systems Holding Co., Ltd, Ningbo, China; 3 College of Mechanical and Energy Engineering, NingboTech University, Ningbo, China; 4 State Key Laboratory in Ultra-Precision Machining Technology, Department of Industrial and Systems Engineering, The Hong Kong Polytechnic University, Hung Hom, Kowloon, Hong Kong; University of Sharjah, UNITED ARAB EMIRATES

## Abstract

The Ti-6Al-4V titanium alloy is a kind of light alloy material with high specific strength, corrosion resistance and heat resistance. Because of its excellent performance, it has become an important material in aerospace industry. However, this kind of alloy has very poor machinability, and rapid tool wear is a very serious problem in titanium alloy processing. At present, it is difficult to guarantee the ultra-precision machining quality of titanium alloy materials, which limits its application in high-tech fields. In order to solve this problem, the influence of cutting speed on ultra-precision cutting process of titanium alloy was analyzed comprehensively. and it was found that better surface quality could be obtained at lower cutting speed. In order to study the influence of cutting speed in ultra-precision cutting of titanium alloys, cutting experiments have been carried out. Additionally, a finite element model was established to analyze the ultra-precision cutting process. Also, the constitutive model, damage model, friction model, and heat transfer in the modeling process were discussed. The chip morphology, cutting temperature, cutting force, and surface morphology under different cutting velocities are analyzed by simulation. Then, the simulation results were compared with the experimental results. The findings show that cutting speed has great influence on the ultra-precision turning of the Ti-6Al-4V alloy and the surface roughness obtained by ultra-precision cutting of titanium alloy can be lower than 20 nm at a lower cutting speed.

## 1. Introduction

Ti-6Al-4V titanium alloy is a kind of (α+β) phase titanium alloy, which has comprehensive mechanical properties and high specific strength. It is widely used in the manufacture of aircraft engine fans, compressor plates and blades, as well as various load-bearing beams, frames, joints and fasteners in aircraft structures. Because of its excellent properties, this alloy has been successfully applied not only in aerospace, but also in medical, chemical industry, petroleum, shipbuilding and other fields [1–3].

With the development of the aerospace industry, in the process of aircraft production, the machining accuracy and surface quality of some key components must be extremely high. Therefore, demand for the ultra-precision machining of titanium alloys is increasing. Thus, the ultra-precision cutting technology of titanium alloy materials has been paid more and more attention in recent years [4–6]. Sakamoto et al [4] tried ultra-precision cutting of a titanium alloy, and achieved a surface roughness less than 100 nm by cutting the titanium alloy with a diamond tool. In addition, Yip et al [5] used a single-point diamond tool to cut the Ti-6Al-4V alloy, and studied the brittle and ductile transition of the material during ultra-precision cutting. Besides, Xiong et al [6] also discussed ultra-precision turning mechanism of the Ti-6Al-4V titanium alloy.

However, due to their low thermal conductivity, high chemical activity and low elastic modulus, titanium alloys are usually considered to be difficult to cut. Mello et al [7] discussed the difficult machinability of different titanium alloys and analyzed the effect of control factors on the response variables was measured using ANOVA. In ultra-precision cutting, especially when diamond tools are used, tool life is extremely short. Therefore, it is difficult to ensure the ultra-precision cutting quality of titanium alloys, which limits the application of titanium alloys in the field of ultra-precision cutting [7–9]. Some researchers have done a lot of work to develop ultra-precision cutting mechanisms for improving the machining quality of titanium alloys [10–13]. Zareena et al [10] studied the wear mechanism of single-crystal diamond tool in the ultra-precision machining of titanium alloys. In addition, Ni et al [11] investigated the effect of material anisotropy on the ultra-precision machining of the Ti-6Al-4V alloy fabricated by selective laser melting. Hu et al [12] used an experimental approach to study the sear of single-crystal diamond tools in the ultra-precision cutting of titanium alloys. Besides, Lou et al [13] also used an electro-pulsing treatment technology to improve the machinability of titanium alloys in ultra-precision machining. In these literatures [14–20], the research shows that the surface roughness of titanium alloy materials obtained by ultra-precision turning is difficult to be lower than 20 nm, and if the surface roughness needs to be lower than 20 nm [14, 15], further ultra-precision polishing [16, 17] or other magnetic auxiliary processing [18] and ultrasonic methods [19] are required.

In recent years, with the development of computer science, finite element technology has been widely used in precision cutting field. This method provides support for the research on chip formation, cutting temperature prediction, microstructure evolution, cutting force and residual stress [21–23]. In addition, finite element technology has become an indispensable means to study the cutting mechanism of titanium alloys [24–32]. Davim et al [23] integrated many of the relevant aspects in the development of coolant assisted finite element method (FEM) simulations applied in difficult-to-cut materials, including titanium alloy. Moola et al [25] applied the finite element model to the machinability of Ti-6Al-4V in dry cutting environment using cubic boron nitride and a polycrystalline diamond tool. Naresh et al [26] proposed the application of elastoplastic model based on explicit finite element analysis to simulate the erosion behavior of titanium alloys in abrasive water jet machining. Wang et al [27] also established a more reliable three-dimensional finite element simulation model, and simulated the milling process in Ti-6Al-4V titanium alloy. Moreover, Mishra et al [28] used three-dimensional finite element model to predict cutting forces when processing titanium alloys under dry conditions.

There are several factors that affect the ultra-precision cutting process of titanium alloys, but in this study, the influence of cutting speed on the cutting process is evaluated by experiments and simulations. A two-dimensional orthogonal finite element cutting model was established, and ultra-precision turning of the Ti-6Al-4V titanium alloy with different cutting speeds is simulated. In addition, the effect of cutting speed on chip morphology, cutting force, cutting temperature and surface morphology of the Ti-6Al-4V alloy are verified through cutting experiments.

## 2. Experimental setup

In this study, the Ti-6Al-4V titanium alloy was used as the workpiece material. The mechanical properties of the alloy are listed in Table 1.

**Table 1:** Physical properties of Ti6Al4V alloy.

Properties	Values
Density	4420 kg/m3
Hardness	HRC 31
Elastic modulus	110 GPa
Yield strength	940 MPa
Thermal conductivity	7.8 W/mk
Poisson’s ratio	0.33

**Fig 1:**
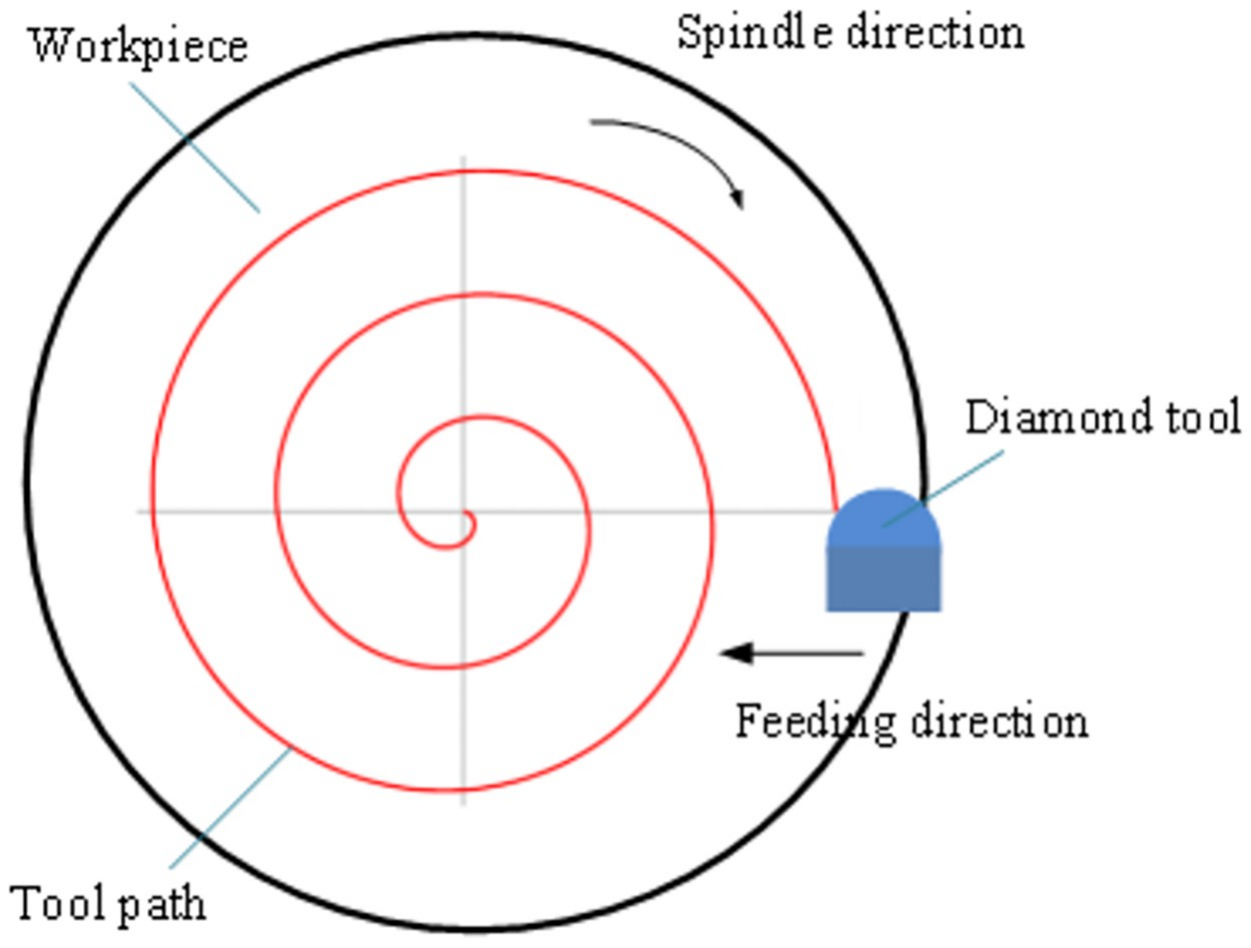
Schematic diagram of the ultra-precision turning process with a single-point diamond tool.

A schematic diagram of ultra-precision turning with single-point diamond tool is shown in Fig 1. When turning the end face, the workpiece is driven by the spindle of the machine tool to rotate, and the turning tool is clamped on the tool holder to move. The cutting speed is the linear speed at the contact point between the machined surface of the rotating workpiece and the turning tool.

**Fig 2:**
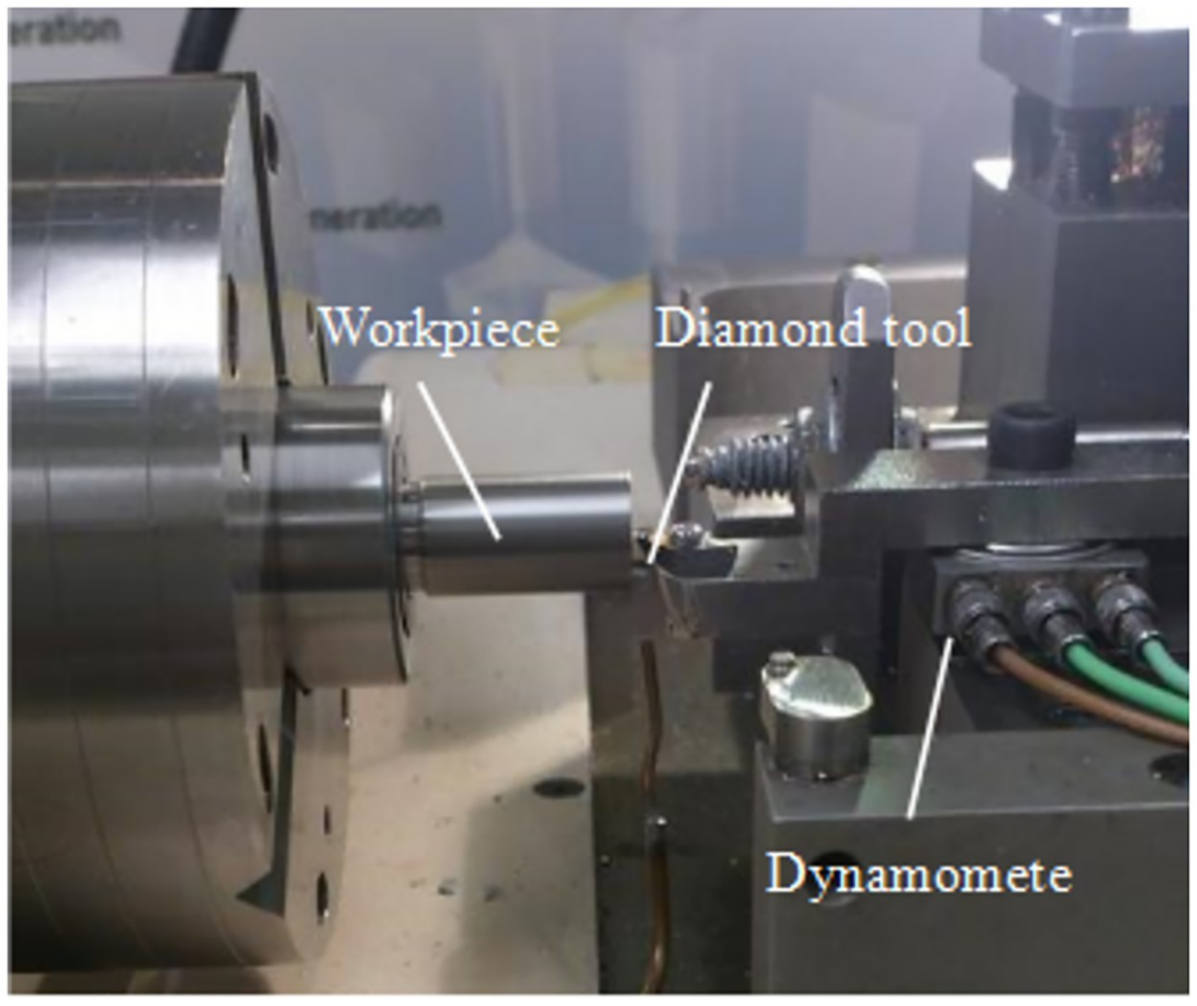
Cutting setup of the Ti-6Al-4V alloy.

The ultra-precision cutting tests of the Ti-6Al-4V alloy were carried out on an Optoform 30 two-axis ultra-precision numerical control machine, and a single-point diamond tool was used in the experiment. The experimental setting of ultra precision cutting is shown in Fig 2. The Ti-6Al-4V samples were cylindrical with a diameter of 14 mm. Additional parameters of the ultra-precision turning process and tool geometry parameters are shown in Table 2. The selection of cutting parameters is based on the reference of these several literature on ultra-precision cutting of titanium alloy [19, 33, 34].

**Table 2:** Cutting parameters of Ti6AL4V alloy.

Parameters	Value
Tool material	Diamond
Tool brand	Contour
Tool radius(mm)	0.5
Tool height (mm)	3.2
Tool rake angle(o)	0
Tool clearance angle (o)	15
Cutting depth (μm)	3
spindle speed (r/min)	1000,2000,3000
Feeding speed (mm/min)	10
Cutting environment	Dry cutting

**Fig 3:**
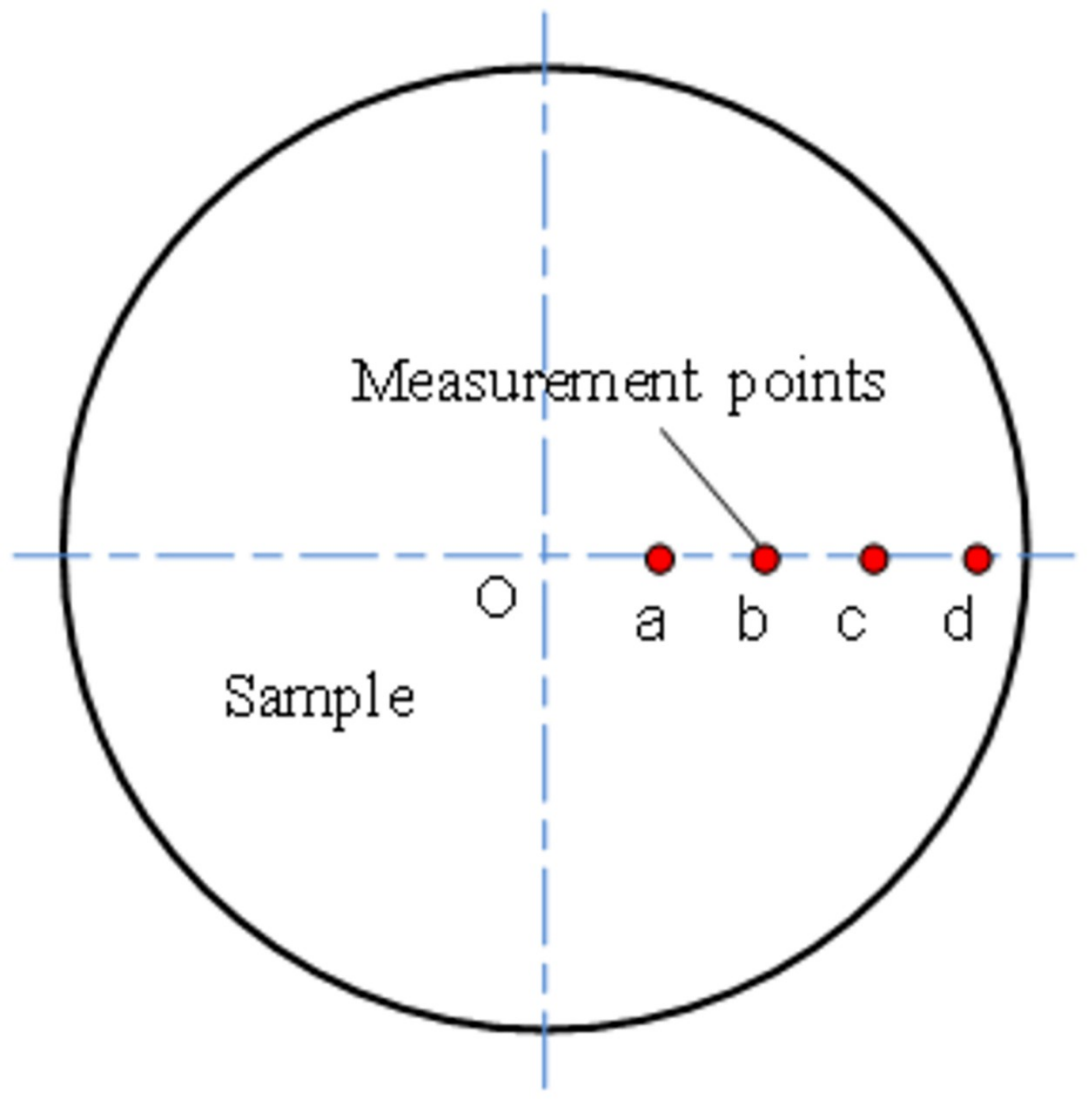
Surface roughness measurement positions.

The surface roughness and surface morphology of the titanium alloy samples were obtained by Wyko NT8000 ultra-precision optical measuring machine. Measure points a, b, c and d as shown in Fig 3, were located 1.5 mm, 3 mm, 4.5 mm, and 6 mm from the center on the right horizontal line of the end face. Under the same spindle speed, the distance from the center of the sample end face is not the same, the cutting line speed is different. The cutting speed at each measuring point can be calculated by using the following equation.

v=ϖr (1)

where *ν* represents the linear speed of each point, *ϖ* denotes the angular velocity of sample rotation, and *r* is the distance from the center of the sample.

### 3. 2D finite element cutting model

**Fig 4:**
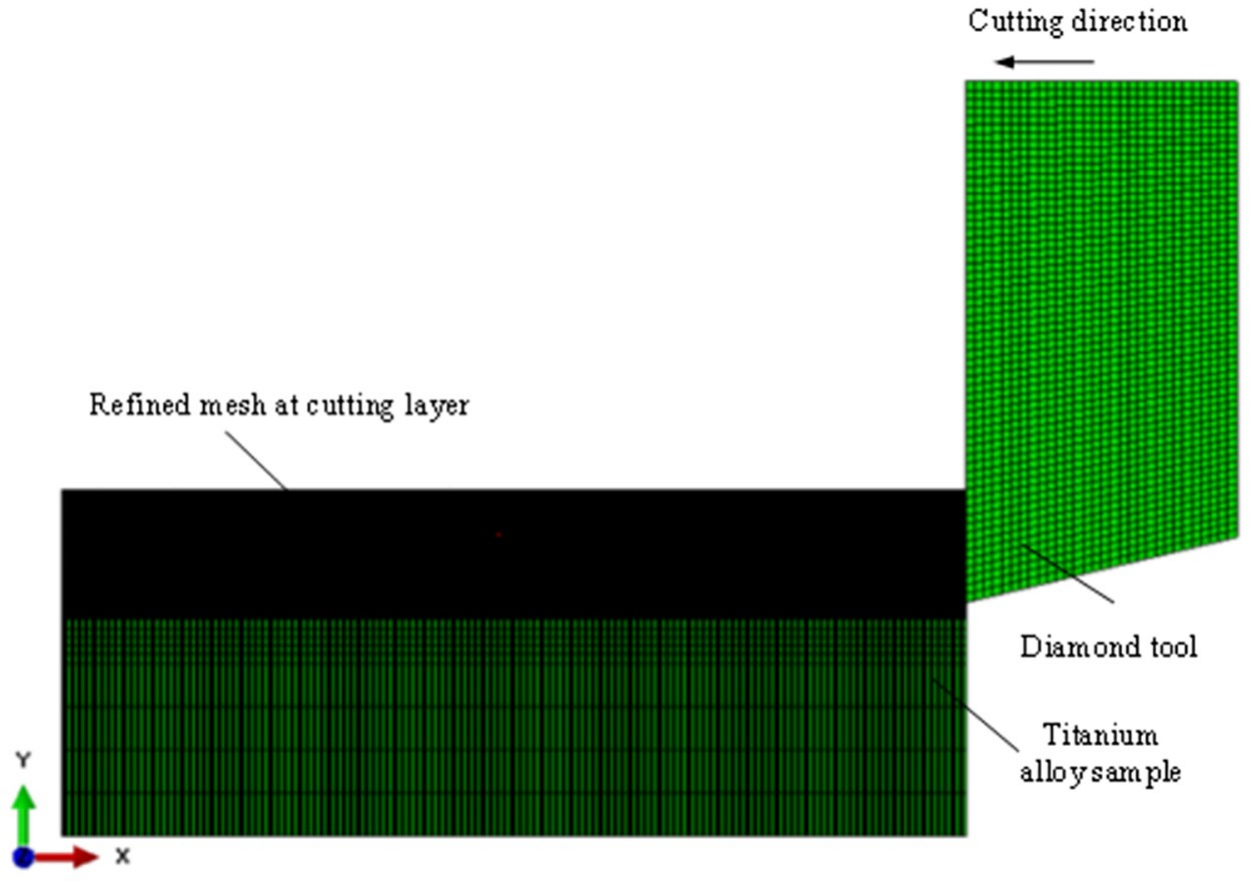
2D finite element model for the Ti-6Al-4V alloy.

Two-dimensional orthogonal cutting model is ideal model to study the cutting mechanism and reveal metal cutting behavior, so it has been widely used in the study of metal cutting process. A Two-dimensional finite element model of the ultra-precision cutting of the Ti-6Al-4V alloy was established by Abaqus software, as shown in Fig 4. In addition, the meshes of the cutting layer of the workpiece were refined to improve the calculations accuracy. During the processing of cutting simulation, the workpiece remained stationary, and a speed constraint was applied to the tool. Due to the low thermal conductivity of the titanium alloy, the adiabatic analysis step was more suitable for solving the cutting process of the Ti-6Al-4V alloy, so its is used for the simulation. The detailed parameters of this model are shown in Table 3. The parameters of the cutting simulation are the same as the parameters of the cutting experiments. A CPE4R mesh type was used and the mesh size of the workpiece was 0.001 μm × 0.001 μm. The initial temperature was 20 °C, and the cutting environment was dry cutting. Besides, the average calculation time for each set of parameters was approximately 72 hours.

**Table 3:** Value of cutting force Fx and surface roughness.

		Point A	Point B	Point C	Point D
1000 r/min	Fx simulation (N)	0.31	0.43	0.54	0.67
	Fx experiment (N)	0.33	0.48	0.59	0.7
	Ra (nm)	18.23	20.27	25.65	29.72
2000 r/min	Fx simulation (N)	0.38	0.51	0.64	0.74
	Fx experiment (N)	0.41	0.54	0.67	0.82
	Ra (nm)	22.34	25.16	27.81	31.60
3000 r/min	Fx simulation (N)	0.44	0.58	0.71	0.86
	Fx experiment (N)	0.47	0.62	0.75	0.92
	Ra (nm)	25.11	29.67	31.04	36.87

The material constitutive model is a mathematical expression of the strength-stress-strain-time relationship, which is very important to metal cutting simulation. Johnson-Cook material model [35] can accurately express the phenomenon of high heat and high strain during metal cutting, so it was also used to simulate the cutting of the Ti-6Al-4V alloy. The stress-strain relationship in the Johnson-Cook material model can be expressed using Eq (2):

$$\sigma =(A+B\varepsilon^{n})(1+C\;\text{ln}\frac{\dot{\varepsilon }}{{\dot{\varepsilon }}_{0}})[1-{\left(\frac{T-T_{r}}{T_{m}-T_{r}}\right)}^{m}$$ (2)

where *ε* is the equivalent plastic strain, $\dot{\varepsilon }$ and ${\dot{\varepsilon }}_{0}$ represent the equivalent and reference plastic strain rates respectively, and *T*, *Tm*, and *Tr* are the material temperature, melting point and initial temperature respectively. In addition, *A*, *B*, *n*, *C* and *m* are the Johnson-Cook coefficients. The five coefficients of the Ti-6Al-4V alloy in the cutting simulation are 1150 Mpa, 870 Mpa, 0.35, 0.22, and 2.15, respectively.

When the Johnson-Cook constitutive model is used, the Johnson-Cook fracture criterion [36] is also necessary to assist chip separation in the simulation. Regarding the damage criterion, when the equivalent plastic strain exceeds a critical point, the damage begins. The Johnson-Cook damage criterion is shown in Eq (3).


$${\dot{\varepsilon }}_{f}=[D_{1}+D_{2}\;\text{exp}(D_{3}\frac{p}{q})][1+D_{4}\;\text{ln}(\frac{\dot{\varepsilon }}{{\dot{\varepsilon }}_{0}})](1+D_{5})$$ (3)

In this equation, D_*1*_-D_*5*_ stand for damage coefficients, p stands for the hydrostatic pressure, q stands for Mises stress, $\dot{\varepsilon }$ stands for the failure strain and ${\dot{\varepsilon }}_{0}$ is the reference strain rate. The five damage coefficients of the Ti-6Al-4V alloy are 0.04, 0.52, 0.3, 0.05, and 2.7, respectively.

In the process of metal cutting, the friction between the tool and the workpiece is very complicated, which directly affects the life and performance of the tool. In metal cutting, especially when machining high-strength workpiece materials, the pressure and temperature produced by friction are the primary reasons of tool failure. The contact area between the chip and the tool can be divided into a sliding region and a sticking region [37]. The sliding region follows Coulomb’s friction law and the critical friction stress is equal to the shear stress in the sticking region. The friction coefficient in the cutting simulation was set at 0.30 and the actual value of the friction coefficient was obtained through the material friction experiments.

In metal cutting, besides the heat generated by friction, the energy consumed by the elastic and plastic deformation of metals under the cutting action of the tool is also an important heat source. In cutting, the heat mainly comes from plastic deformation and friction. Plastic deformation mainly occurs in the primary shear zone, and most of the deformation energy is transformed into heat energy during cutting. For the cutting simulation of the Ti-6Al-4V alloy, it is assumed that 90% of the deformation energy is converted to heat.

## 4. Results and discussions

### 4.1 Analysis of surface quality

**Fig 5:**
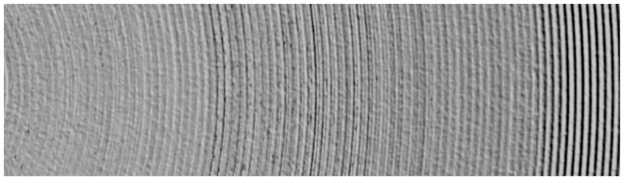
Surface morphology of the titanium alloy sample after turning.

Fig 5 shows the surface topography of the Ti-6Al-4V titanium alloy obtained by ultra-precision turning. It is difficult to obtain satisfactory surface quality through ultra-precision turning, so there are a lot of defects on the surface, such as burrs and pits.

**Fig 6:**
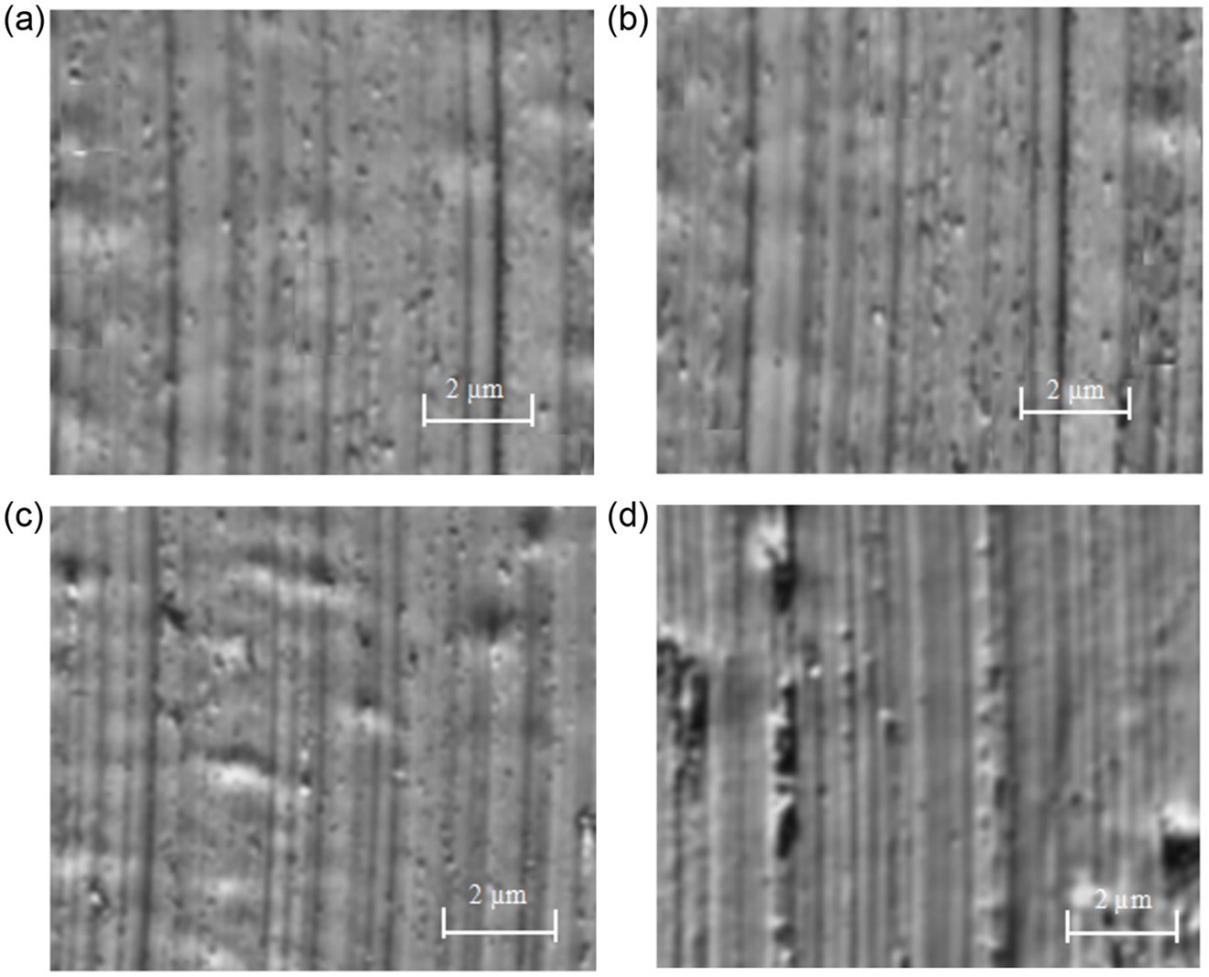
Surface morphology of four points under spindle speed 3000 r/min. (a) Surface morphology of point a. (b) Surface morphology of point b. (c) Surface morphology of point c. (d) Surface morphology of point d.

SEM images of four different points (a, b, c and d) on the upper surface of the titanium alloy sample are shown in Fig 6. The results shows that with the increase of the distance from the sample center, more defects appear on the surface. In addition, higher cutting speeds leads to poor surface quality. Therefore, in the ultra-precision machining of titanium alloys, in order to obtain good surface quality, it is preferred to use a lower cutting speed.

**Fig 7:**
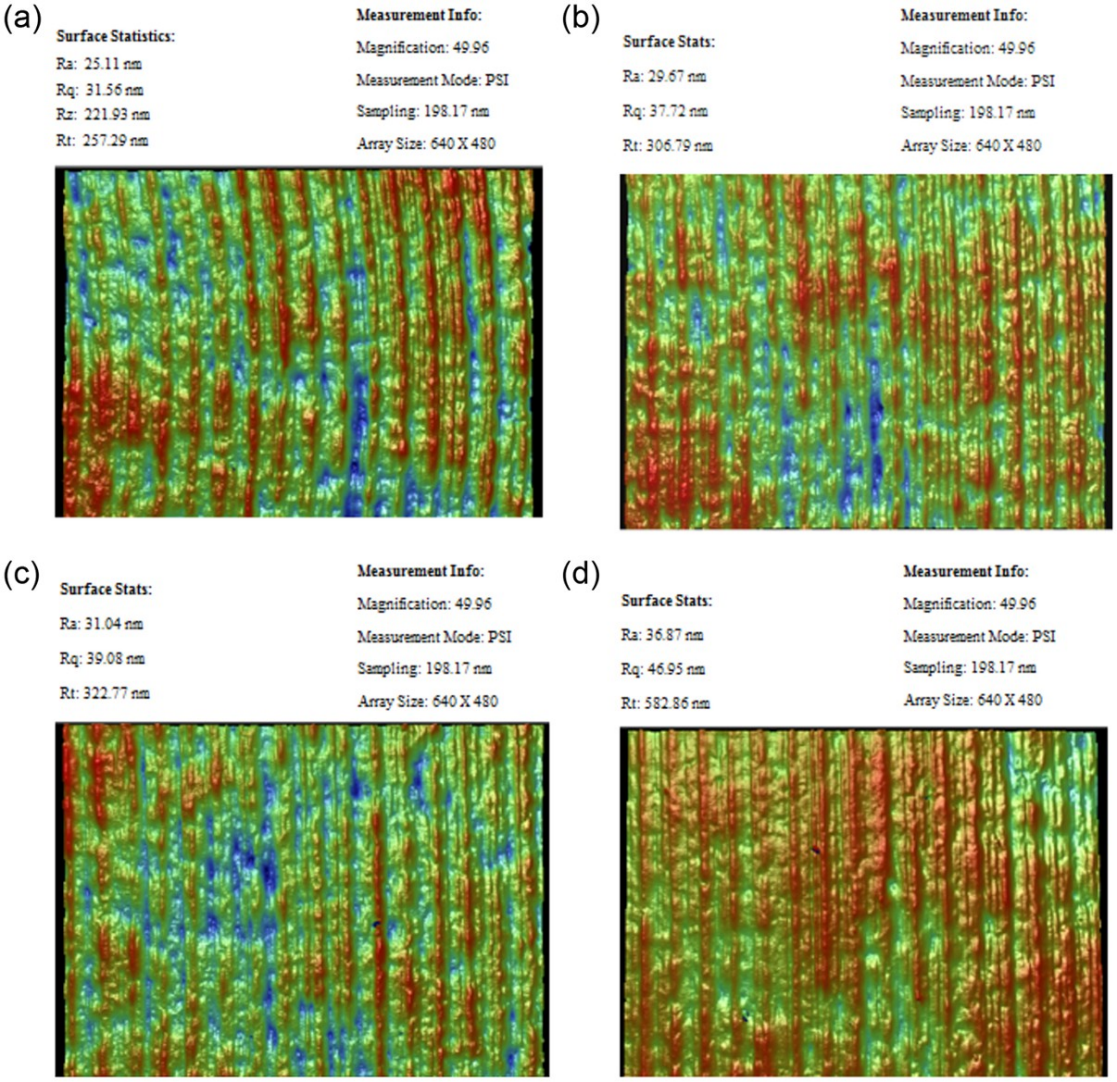
Surface morphology at the four points under spindle speed 3000 r/min. (a) 3D surface morphology of point a. (b) 3D surface morphology of point b. (c) 3D surface morphology of point c. (d) 3D surface morphology of point d.

**Fig 8:**
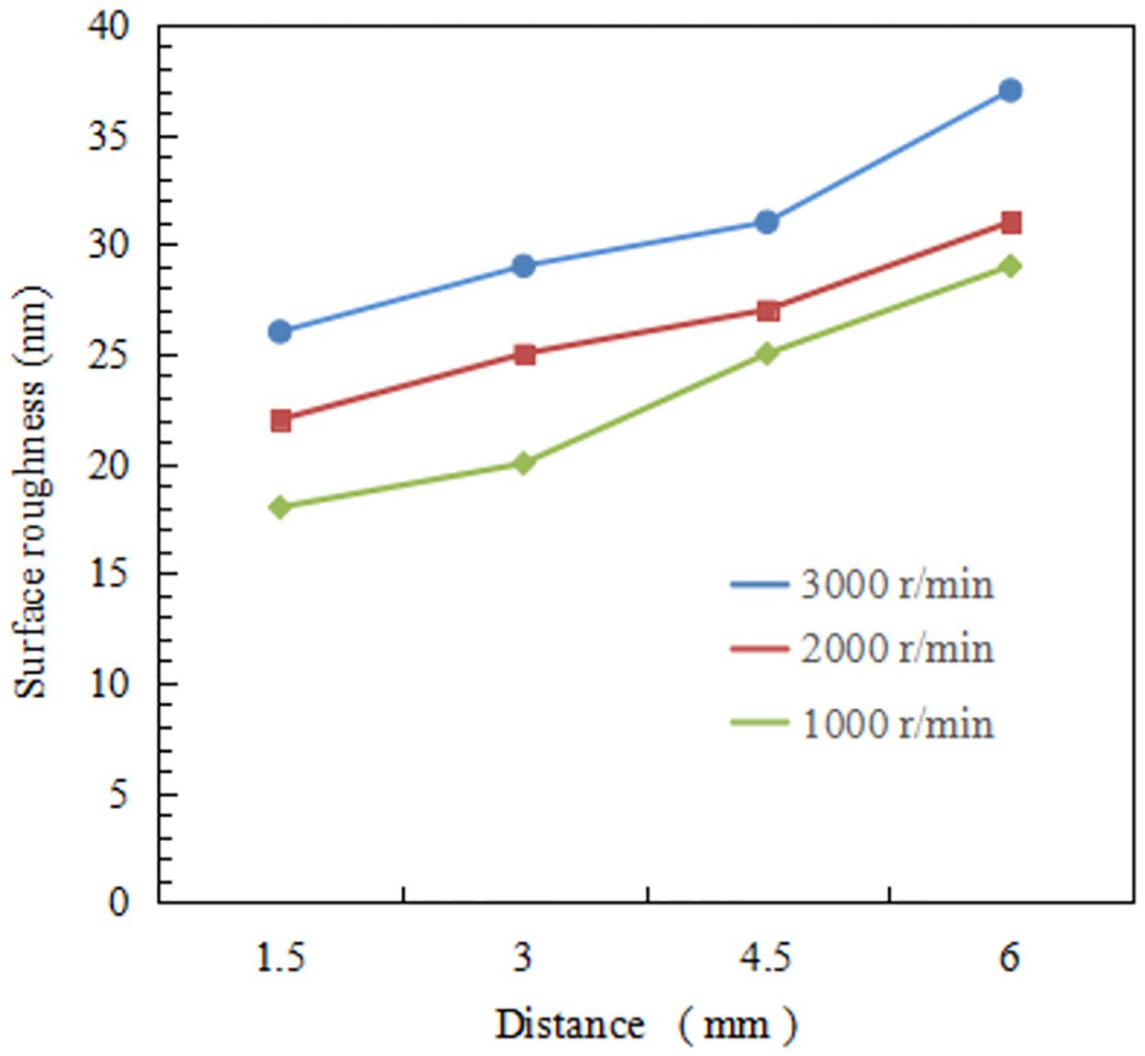
Average surface roughness values of four titanium alloy samples under different cutting speed.

Fig 7 shows the surface roughness values and surface topography at the four points obtained by ultra-precision optical test equipment. The figure indicates that the surface roughness value rises with increasing distance from the center. This proves that the end surface quality after turning is affected by an increase in cutting speed. In order to confirm this discovery, five titanium alloy samples were used with the same cutting parameters and measured the surface roughness at the same position. The average surface roughness of four different Ti-6Al-4V titanium alloy samples is shown in Fig 8 and Table 3. It can be seen that the surface roughness value keeps increasing and surface quality gradually deteriorates with the increase of cutting speed. At point d, the surface roughness exceeds 35 nm. In addition, as can be seen from the figure, the farther away from the center of the sample, the greater the roughness value of the machined surface. This is because the farther away from the center of the sample, the greater the linear velocity of the contact point between the workpiece and the tool, the stronger the cutting effect, so the greater the cutting force and the higher the cutting temperature, which will affect the surface quality. In addition, the farther away from the center of the sample, the greater the cutting force, the greater the impact on the vibration of the machine tool system, and the intensified vibration will also lead to the decline of surface quality. The results show that the surface roughness of point a obtained by ultra-precision cutting of titanium alloy under the spindle speed 1000 r/min can be lower than 20 nm.

### 4.2 Analysis of chip morphology

**Fig 9:**
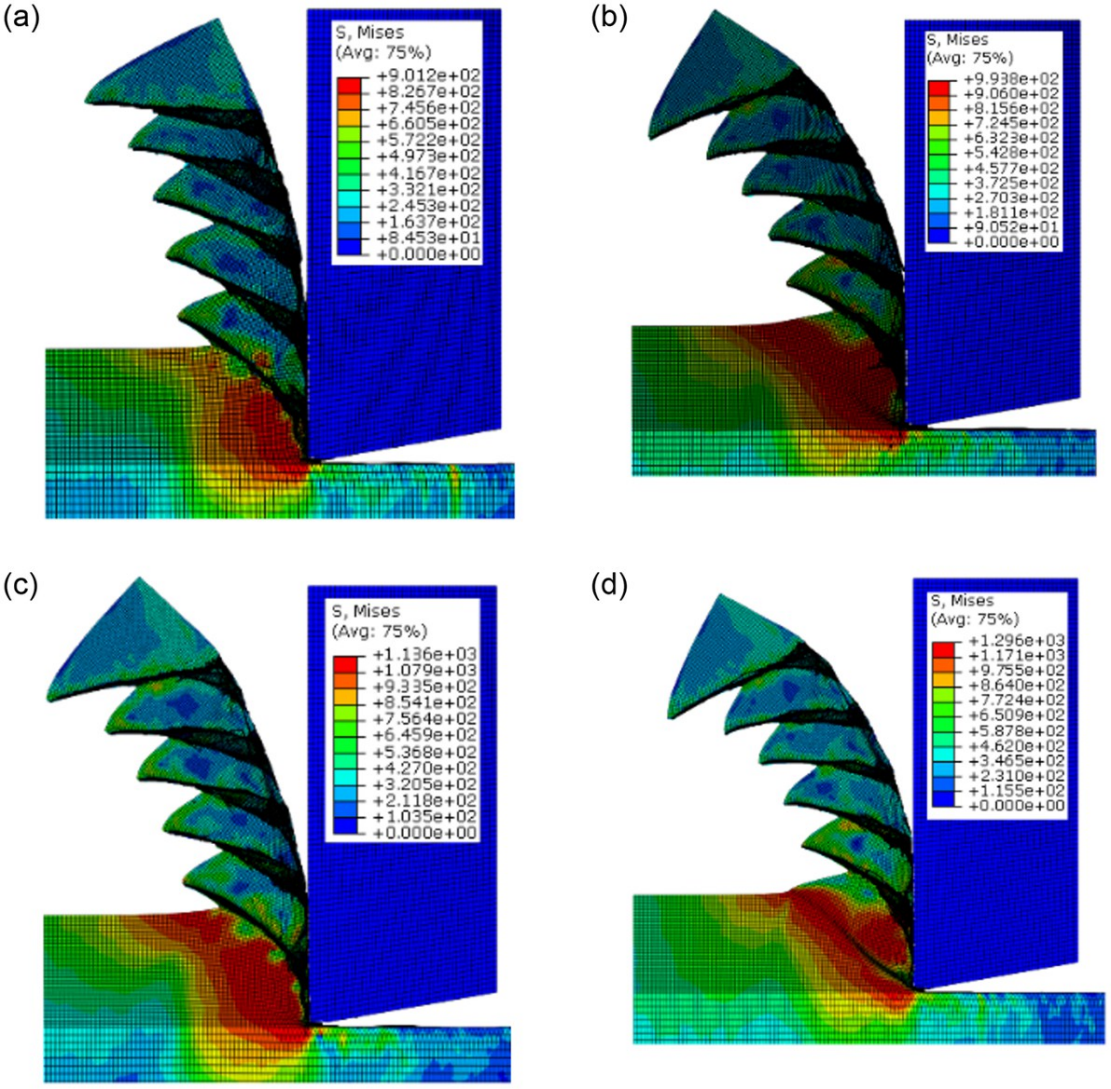
Chip formation at the different points under spindle speed 3000 r/min. (a) Cutting speed at point a. (b) Cutting speed at point b. (c) Cutting speed at point c. (d) Cutting speed at point d.

In metal cutting, the process of chip formation is very complicated, and there are several factors that affect shape of chip. Under different cutting parameters, the chip formation mechanism is different and the chip presents different morphology. Fig 9 shows the chip morphology of the Ti-6Al-4V titanium alloy at points a, b, c and d obtained by simulation at different cutting speeds. At all four cutting speeds, the chip shape is serrated, and the cutting speed has no obvious effect on the sawtooth shape. The cutting speed increased and the strain rate increased, while titanium alloy materials have a strong strain strengthening effect, so the stress in the shear zone increased when cutting speed increased. But the chip shape did not change significantly and the shape of the chips was serrated. This is because the thermal conductivity of Ti-6Al-4V alloy is very low, which makes it difficult to release the cutting heat in the shear zone quickly, which leads to the chip of titanium alloy material serrated due to the thermal softening effect caused by the increase of cutting temperature at a wide cutting speed range.

**Fig 10:**
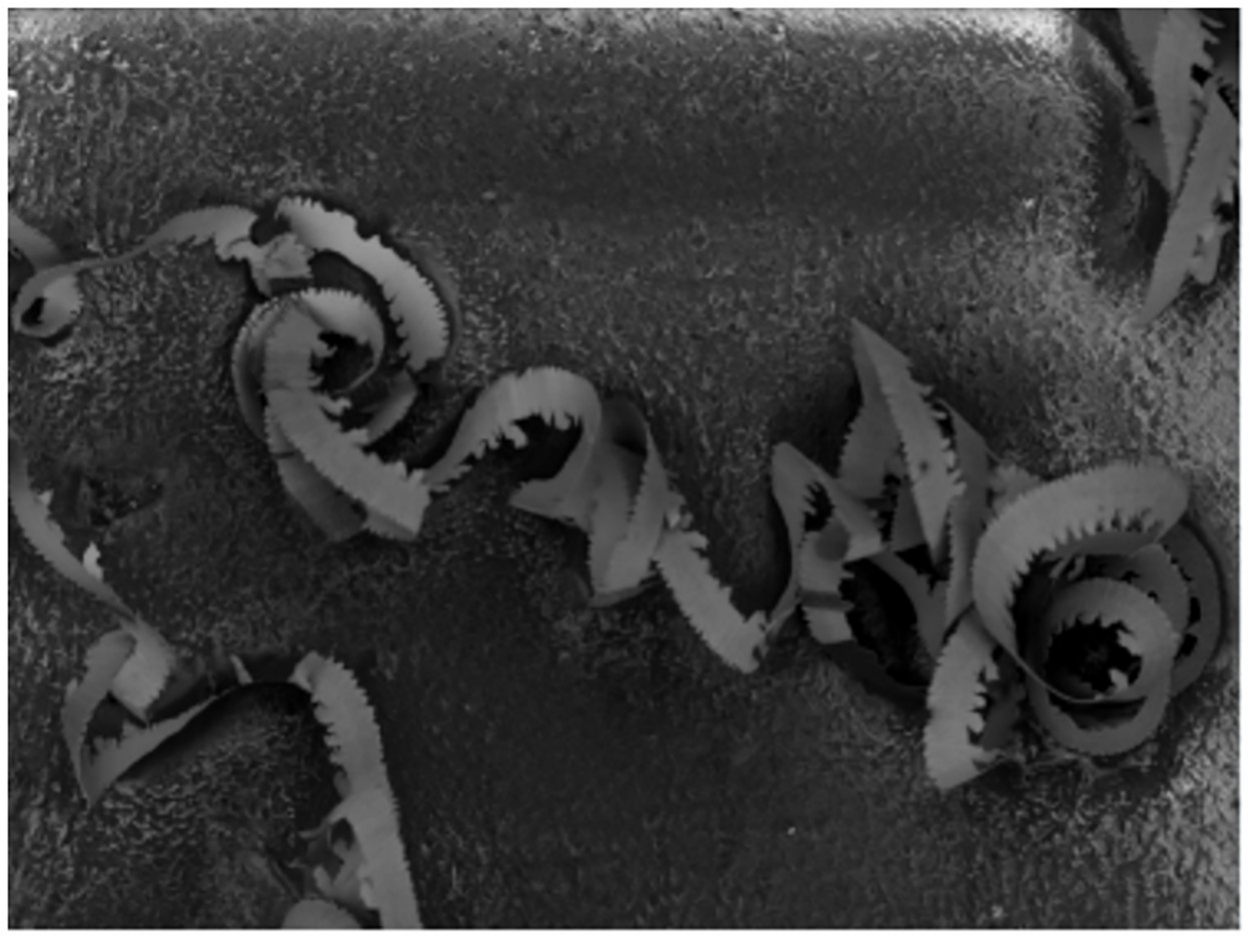
Chip obtained by ultra-precision turning.

**Fig 11:**
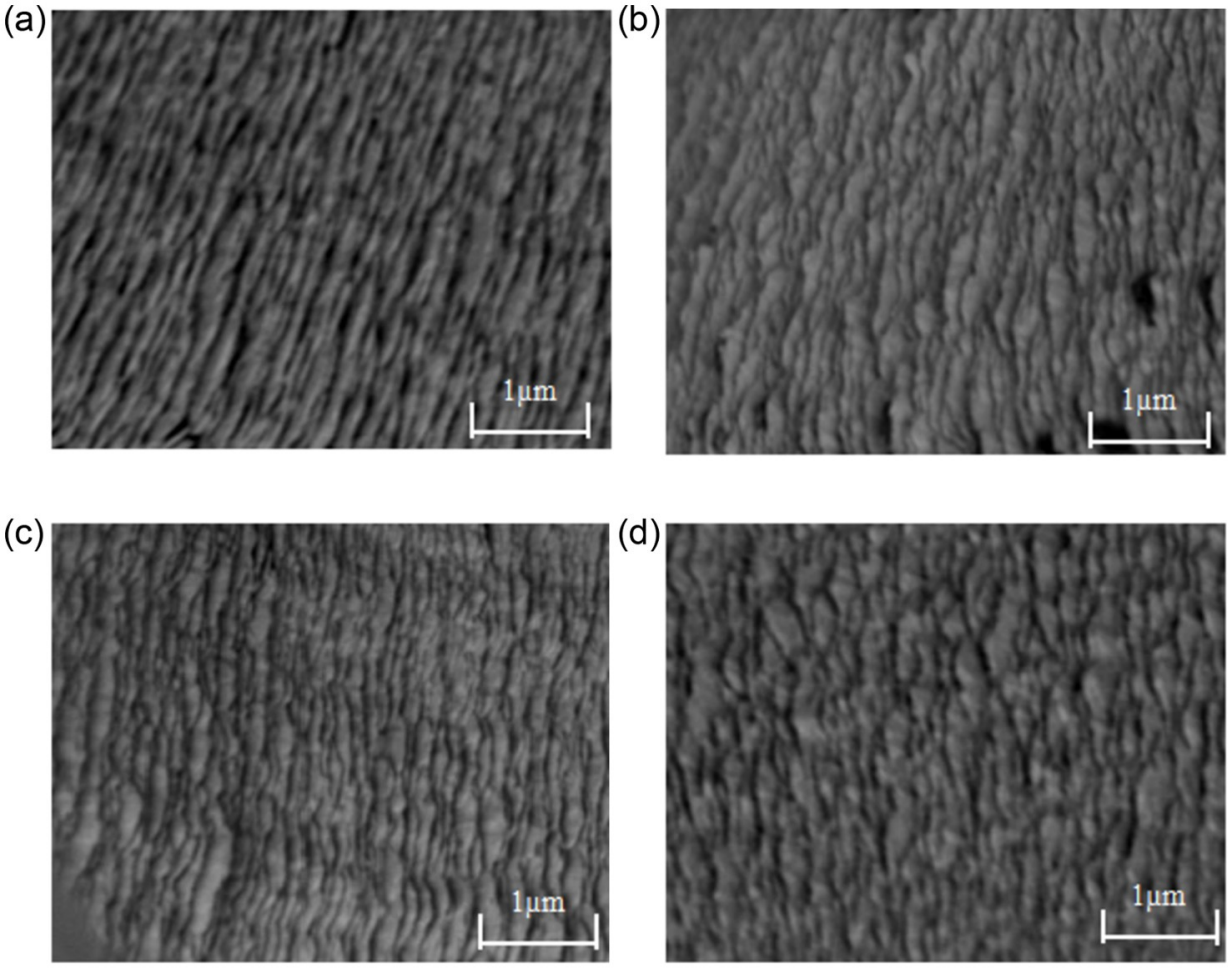
Chip morphology under the different measured points under spindle speed 3000 r/min. (a) Chip surface obtained near point a. (b) Chip surface obtained near point b. (c) Chip surface obtained near point c. (d) Chip surface obtained near point d.

The chip obtained from the end-face turning of titanium alloy in cutting experiment are shown in Fig 10. Also, Fig 11 presents the enlarged morphology at four different positions on the chip. These figures show that the chip obtained through ultra-precision cutting of the titanium alloy are serrated. Therefore, the shape of the experimental chip is consistent with the results of the numerical simulation. At the same time, it is also proved that the cutting speed has no obvious influence on the shape of titanium alloy chips.

### 4.3 Influence on cutting force

**Fig 12:**
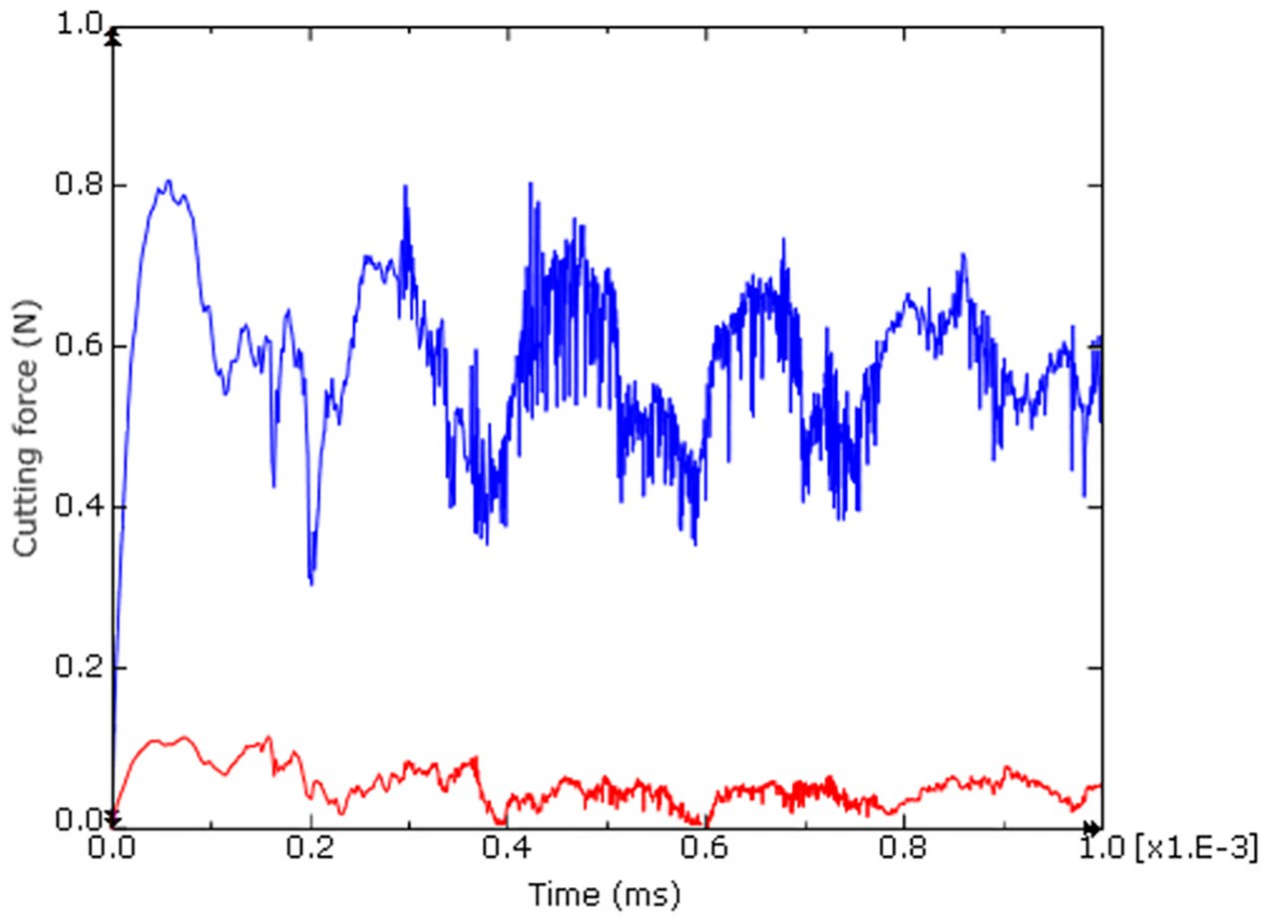
Simulated cutting force curve at point a under spindle speed 3000 r/min.

**Fig 13:**
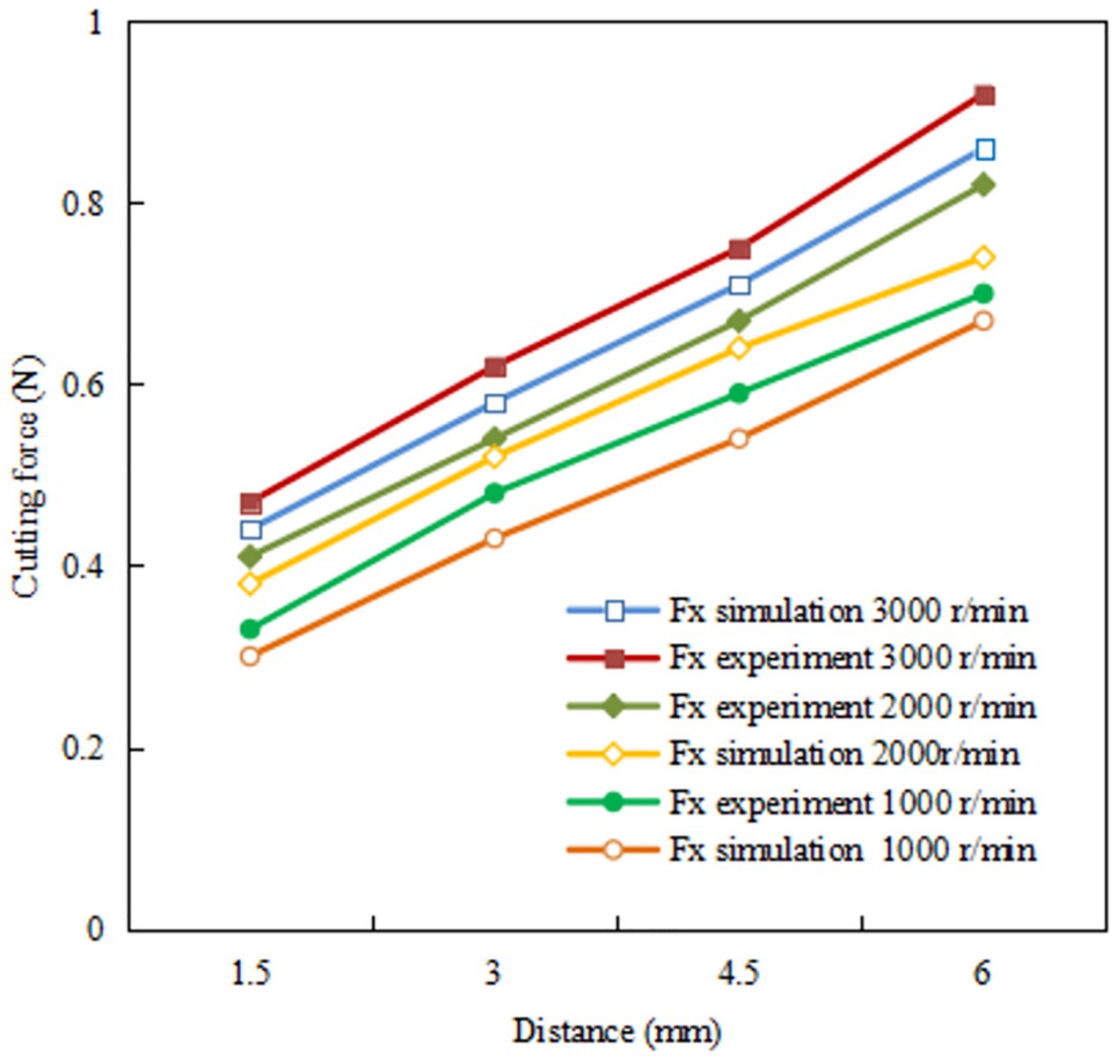
Comparison of cutting forces between the simulation and the experiment.

Cutting force is the resistance of cutting tool to the workpiece material, which can be influenced by many factors. Cutting force curves Fx and Fy of the Ti-6Al-4V alloy achieved through simulation of ultra-precision cutting are shown in Fig 12. The fluctuation of cutting force in the process of the serrated chip formation is revealed. The period of cutting force fluctuation corresponds to the formation period of serrated chips. Fig 13 and Table 3 shows a comparison of the primary cutting force Fx between the simulation and the experiment. In addition, it can be seen from the figure that the farther away from the center of the end face of the sample, the greater the linear speed, the greater the cutting force.

## 5. Conclusions

In order to discuss the influence of different cutting speeds on ultra-precision turning of titanium alloys, the cutting mechanism was determined by finite element technology. In this study, an orthogonal finite element model was presented. The numerical values for stress distribution, chip shape, cutting force and cutting temperature were obtained through simulation.

1. The properties of the chips in the simulation were consistent with those of the chips obtained by the cutting tests. The chip shape is sawtooth in both cutting experiment and numerical simulation.
2. The influence of different cutting speeds on the surface quality and cutting force of the Ti-6Al-4V alloy was studied by a comparison between experiment and simulation.
3. The results of this study confirm that cutting speed has a great influence on ultra-precision turning of the Ti-6Al-4V alloy, especially on surface roughness. And the surface roughness of titanium alloy can be lower than 20 nm by selecting suitable cutting speed.

## References

[1] Davim JP. Machining of titanium alloys. Berlin, Heidelberg: Springer-Verlag;2014.ISBN 978-3-662-43901-2

[2] Davim J. Paulo Machining: fundamentals and recent advances, Springer, 2008, ISBN: ISBN 978-1-84800-212-8

[3] Carvalho Silvia & Horovistiz Ana & Davim J. (2022). Morphological characterization of chip segmentation in Ti-6Al-7Nb machining: A novel method based on digital image processing. Measurement. 206. 112330. doi: 10.1016/j.measurement.2022.112330

[4] Sakamoto S, Shinozaki A, Yasui H. Possibility of Ultra-Precision Cutting of Titanium Alloy with Diamond Tool[C]// The Twentieth Annual Meeting of the American Society for Precision Engineering. Dept. of Mechanical Engineering & Materials Science, Kumamoto University, 2-39-1, Kurokami, Kumamoto, 860–8555, Japan, 2005.

[5] Yip WS, To S (2018) Ductile and brittle transition behavior of titanium alloys in ultra-precision machining. Sci Rep 8(1):3934. doi: 10.1038/s41598-018-22329-2 29500386 PMC5834465

[6] Xiong R, Wu H. Study on cutting mechanism of Ti6Al4V in ultra-precision machining. International Journal of Advanced Manufacturing Technology, 2016, 86(5–8):1–7.

[7] Mello A O, Pereira R B D, Lauro C H, et al. Comparison between the machinability of different titanium alloys (Ti–6Al–4V and Ti–6Al–7Nb) employing the multi-objective optimization[J].Journal of the Brazilian Society of Mechanical Sciences and Engineering, 2021, 43(11):1–14

[8] Zhang Yuanliang, Zhou Zhimin, Wang Jinlong, et al. Diamond Tool Wear in Precision Turning of Titanium Alloy. Advanced Manufacturing Processes, 2013, 28(10):1061–1064.

[9] Heiji, Yasui, Akira, et al. The Ultra-Precision Cutting of Titanium Alloy with Coated-Cemented-Carbide Tool[J]. Proceedings of JSPE Semestrial Meeting:62–62.

[10] Zareena A. R., Veldhuis S. C., 2012, Tool wear mechanisms and tool life enhancement in ultra-precision machining of titanium, Journal of Materials Processing Technology 212(3): 560–570.

[11] Ni Chenbing, Zhu Lida, Zheng Zhongpeng, Zhang, et al. Effect of material anisotropy on ultra-precision machining of Ti-6Al-4V alloy fabricated by selective laser melting. Journal of Alloys and Compounds.2021.848:156457-

[12] Hu L H, Zhou M. Experimental Study on the Wear of Single Crystal Diamond Tools in Ultra-Precision Cutting of Titanium Alloy. Key Engineering Materials, 2016, 693:1015–1021.

[13] Lou YG, Wu HB. Improving machinability of titanium alloy by electro-pulsing treatment in ultra-precision machining. International Journal of Advanced Manufacturing Technology, 2017, 93(5–8):2299–2304.

[14] Wu H B, To S. Effects of electropulsing treatment on material properties and ultra-precision machining of titanium alloy. International Journal of Advanced Manufacturing Technology, 2016, 82(9–12):2029–2036.

[15] Kwak N S, Kim J Y, Park D G. A Research on Ultra Precision Machining for Ti-6AL-4V Alloy Based Biomedical Applications Using Nanopositioning Mechanism. Journal of Nano Research, 2013, 25(12):17.

[16] Xu Z, Hongbing W. Influence of path on the ultra-precision polishing process of titanium alloy Ti6Al4V. International Journal of Advanced Manufacturing Technology, 2018, 98(5–8):1–8.

[17] Lou Yonggou, Wu Hongbing, Effect of parameters on surface roughness during the ultra-precision polishing of titanium alloy, PLOS ONE,2022, 17(8): e0272387. doi: 10.1371/journal.pone.0272387 35913977 PMC9342747

[18] Yip Wai Sze, To Sandy, Sustainable Ultra-Precision Machining of Titanium Alloy Using Intermittent Cutting, International Journal of Precision Engineering and Manufacturing-Green Technology,2020, 7,:361–373

[19] Pei Lei & Wu Hongbing. (2019). Effect of ultrasonic vibration on ultra-precision diamond turning of Ti6Al4V. The International Journal of Advanced Manufacturing Technology. 103.

[20] Xiaohua Qian, Xiongying Duan, Jiyan Zou. Effects of different tool microstructures on the precision turning of titanium alloy TC21. The International Journal of Advanced Manufacturing Technology. (2020).106(6), 5519–5526

[21] Davim, J. Paulo, Finite Element Method in Manufacturing Processes, Wiley, 2011, ISBN: 978-1-848-21282-4

[22] Hanjing Lu, Yuanyuan, et al. Dynamics Modelling and Simulating of Ultra-precision Fly-Cutting Machine Tool. International Journal of Precision Engineering and Manufacturing, 2020, 21(2):189–202.

[23] Davim J. & Maranhão C. A study of plastic strain and plastic strain rate in machining of steel AISI 1045 using FEM analysis. Materials & Design. 2009.30. 160–165.

[24] Silvia R C, Lauro C H, Ana H, et al. Development of FEM-based digital twins for machining difficult-to-cut materials: A roadmap for sustainability[J].Journal of manufacturing processes, 2022(Mar.):75.

[25] Moola Reddy, Mohan Kumar, Kumaraesan Shanmugam. (2018). Finite element analysis and modeling of temperature distribution in turning of titanium alloys. Metallurgical and Materials Engineering. 24. 59–69

[26] Naresh Kumar, Shukla Mukul Dr. (2012). Finite element analysis of multi-particle impact on erosion in abrasive water jet machining of titanium alloy. Journal of Computational and Applied Mathematics. 236. 4600–4610.

[27] Wang Zhenda, Ze Xiangbo, Yousuf Yusuf et al. (2021). Three-dimensional finite element simulation of high speed milling of titanium alloy Ti6Al4V. Journal of Physics: Conference Series. 1948. 012130.

[28] Sarvesh Mishra, Sudarsan Ghosh, Aravindan S. (2018). 3D finite element investigations on textured tools with different geometrical shapes for dry machining of titanium alloys. International Journal of Mechanical Sciences. 141. 424–449.

[29] Xiaoling Zhu, Li Cai, Xiaohua Qian. Influence of different cutting edges caused by tool wear on cutting process of titanium alloy TC21 based on finite element model. Proceedings of the Institution of Mechanical Engineers, Part B: Journal of Engineering Manufacture. 2022.237(1–2).

[30] Qian Xiaohua, Duan Xiongying. Constitutive Model and Cutting Simulation of Titanium Alloy Ti6Al4V after Heat Treatment. Materials. 2019.12(24).4145 doi: 10.3390/ma12244145 31835657 PMC6947600

[31] Hu KM, Lo S L, Wu HB, To Sandy. Study on Influence of Ultrasonic Vibration on the Ultra-Precision Turning of Ti6Al4V Alloy Based on Simulation and Experiment. IEEE Access, 2019:1–1.

[32] Aydn M. Numerical study of chip formation and cutting force in high-speed machining of Ti-6Al-4V bases on finite element modeling with ductile fracture criterion. International Journal of Material Forming, 2021. 14:1005–1018.

[33] Sze Wai, Yip & To Sandy. (2019). Sustainable Ultra-Precision Machining of Titanium Alloy Using Intermittent Cutting. International Journal of Precision Engineering and Manufacturing-Green Technology. 7.

[34] Manjunath Karanam & Tewary Suman & Khatri Neha & Cheng Kai. (2022). Precipitation effect on Surface roughness at Ti-6Al-4 V ELI alloy during Ultra-Precision Machining. International Journal on Interactive Design and Manufacturing. 1–9.

[35] Johnson R, Cook WK (1983) A constitutive model and data for metals subjected to large strains high strain rates and high temperatures. The 7th International Symposium on Balistics, The Hague: pp.541-547.

[36] Johnson GR, Cook WH (1985) Fracture characteristics of three metals subjected to various strains, strain rates, temperatures and pressures. Eng Fract Mech 21: 31–48.

[37] Zorev NN (1963) Inter-relationship between shear processes occurring along tool face and shear plane in metal cutting. Int Res Prod Eng, ASME: 42–49.

